# Evidence-Based Status of Forest Healing Program in South Korea

**DOI:** 10.3390/ijerph181910368

**Published:** 2021-10-01

**Authors:** Sujin Park, Soojin Kim, Geonwoo Kim, Yeji Choi, Eunsoo Kim, Domyung Paek

**Affiliations:** 1 Forest Policy and Economics Department, Forest Welfare Division, National Institute of Forest Science, Seoul 02455, Korea; snowshoe@korea.kr (S.P.); kimsoojinsj@korea.kr (S.K.); bkim5020@korea.kr (G.K.); usmile.choi@gmail.com (Y.C.); euncarp2@gmail.com (E.K.); 2 Department of Environmental Health Sciences, Graduate School of Public Health, Seoul National University, Seoul 08826, Korea; 3 Institute of Health and Environment, Seoul National University, Seoul 08826, Korea

**Keywords:** forest healing, forest healing program, forest therapy, meta-analysis, systematic review

## Abstract

Various effects of forest healing on health have been reported, but a certification system to assess the effectiveness of forest healing programs does not exist. In this study, a systematic review (SR) on the “health benefits of forests” and “meta-analysis of forest therapy” was conducted after analyzing the status and level of evidence of 75 forest healing programs that were conducted post-certification in South Korea. The SR for “health benefits of forests” distinguished between activities and time, resulting in 90.9% of walking activities for more than an hour under psychological health, and 100.0% of exercise activities for less than an hour under physiological health. However, the effect of indirect activities performed for more than an hour was unknown. Thus, we confirmed that many indoor activities in the field had low effect size or no established basis regarding the feasibility of its operation. The SR on “meta-analysis of forest therapy” to check whether the program was effective. The highest number of healing effects were obtained for blood pressure (32), followed by psychological depression (24). The findings of this can serve as baseline data to facilitate future development and dissemination of evidence-based forest healing programs.

## 1. Introduction

In 2018, the public value of forests in $186 million, of which $1.52 million (8.3%) was contributed by forest recreation function, which was the fourth highest contributor. This contribution had increased by $574,761 compared to $1.43 million in 2014, whereas the value contributed by “forest healing” increased from $2.02 million in 2014 to $4.30 million in 2018 [1]. With the ongoing increase in public interest in forest recreation and healing, the number of operational therapeutic forests with forest healing programs have increased. These therapeutic forests are forests (including natural and man-made facilities) that are designed for enhancing health, wellness, and happiness—i.e., healing [2].

**Figure 1:**
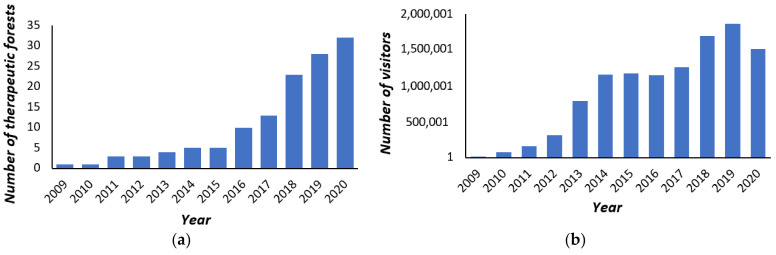
(**a**) The number of therapeutic forests; (**b**) the number of visitors to therapeutic forests.

The Korea Forest Service has been creating and operating therapeutic forests in Korea since opening the first therapeutic forest in 2009, called *San-eum*, which means “the shade of the mountain”. Since then, the total number of national and public therapeutic forests steadily increased to 5 by 2015 and 32 by 2020 (Figure 1). In line with this increasing number of therapeutic forests, the number of visitors to these forests to experience forest healing have also increased rapidly. For example, the number of visitors was merely 1067 in 2009, but increased to 1.7 million in 2015, 1.8 million in 2019, and 1.5 million in 2020 (global pandemic year), indicating that more and more people are visiting therapeutic forests to experience the effects of forest healing.

**Figure 2:**
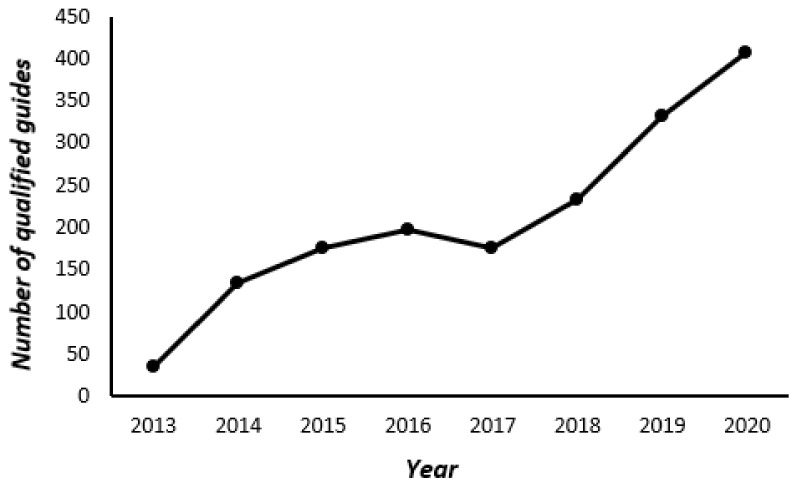
The number of new forest healing guidance certificates (Levels 1 and 2).

Forest healing is not an act of treating diseases, but it is a healing activity that helps in maintaining health and increasing immunity; it reduces stress [3,4,5,6,7,8,9,10,11,12], recovers cognitive functions [13,14,15,16,17,18], and reduces depression [5,12,14,15,16,17,18,19] and cardiovascular disease [20,21,22,23,24,25,26]. As more people visit therapeutic forests to enjoy the benefits and participate in forest healing programs, the number of qualified healing forest guides (instructors) have also been increasing (Figure 2) [27].

The forest healing program was developed based on the gender, age, occupational characteristics, diseases of the participants, and their purpose for visiting. After examining the therapeutic potential and spatial aspects of a site, programs are designed based on different healing factors and treatments. Forest healing factors include the landscape, phytoncides, anions, sounds, sunlight, and oxygen levels [28], whereas the six major therapies of forest healing include plant therapy, water therapy, diet therapy, psychotherapy, climate therapy, and exercise therapy [29].

Generally, a performance assessment determines the success of forest healing programs and also assesses any underlining reasons for changes to the program [30]. However, since standard procedure for forest healing is not available, this assessment is based on the healing efficacy of the program during the development phase; the program is initially run without the requirement of any verification.

To reduce the instances of poor implementation, the Korea Forest Service is validating the forest healing program based on the criteria for the forest education program. However, as the purpose of the forest education and forest healing differs, using this certification system for verification is limited. Therefore, a basis for evaluating the composition and effectiveness of the forest healing program is needed to provide high-quality forest healing programs. Several previously published studies have analyzed the activities of forest healing programs, but these studies did not solely focus on the certified programs; furthermore, the number of programs analyzed was small [31,32,33].

To address previous limitations, this study aimed to analyze 75 forest healing programs that are certified by the Korea Forest Service and the Korea Forest Welfare Promotion Agency (as of December 2020) to validate their activities. This study utilized the certification system of forest education programs conducted by the Korea Forest Service, the Ministry of Gender Equality and Family, and the Ministry of Environment (Table 1).

To establish the basis for the healing programs, the effectiveness of the programs were verified through clinical trials. However, there are difficulties in verifying the effectiveness of all diversifying subjects and programs through clinical trials, and in order to find a method to verify the effectiveness of the program that can be used continuously in the future, this study analyzes the operation status of forest healing programs operated in the field. It was attempted to verify the effectiveness of the program by reflecting the results of previous research. Through the results of this study, it is intended to provide basic data for evaluating forest healing programs in the future.

## 2. Materials and Methods

### 2.1. Research Methods

To determine the current status of forest healing programs, 75 forest healing programs that are operated by the Korea Forest Welfare Institute, the main body of forest welfare, were analyzed. Under these 75 programs, there were 278 detailed programs, out of which 268 were included in the present study after excluding the overlapping programs. Therefore, a total of 268 detailed programs were analyzed and then classified into six categories by referring to the contents written by the forest healing instructor who developed the programs (Table 2).

The participants of the forest healing program were divided according to two classifications: disease states and target groups, with the categories of disease states being normal, chronic, addictive, and environmental diseases. Accordingly, these conditions included the absence of disease (normal); continuous, potential, and pathological conditions (chronic diseases) [33]; substance poisoning such as alcohol, nicotine, and behavioral addiction, which are mainly discussed in the forest (addictive diseases); and subjects with health disorders caused by environmental factors, such as physical, chemical, and biological factors, which include atopic dermatitis and allergies that are mainly experienced in forests (environmental diseases). In addition, the classification of subjects identified the target groups according to their ability to participate in the program activities as infants, adolescents, adults, the elderly, pregnant women, and families.

The classification of the six major therapeutic approaches is based on a previous study [29]; the different approaches include plant therapy, water therapy, diet, psychotherapy, climate therapy, and exercise therapy. These activities were categorized as: walking and hanging around in the forest to improve health (plant therapy); water activities such as walking in the valley or immersing one’s arms and legs in water (water therapy); eating activities such as drinking tea (diet); meditation and contemplation in forests (psychotherapy); activities that utilize the microclimate elements of forests, such as morning and night walks (climate therapy); and activities that include forest scenery and terrain such as forest sports events and trekking (exercise therapy).

Activities for the senses are divided into five categories: visual, olfactory, auditory, tactile, and palate. These subdivisions can be understood as: programs that utilize color perception (visual); programs that utilize fragrances such as herbs, flowers, and fruits (olfactory); programs where participants listen to the wind and leaf swaying sounds from nature (auditory); programs where participants touch petals, leaves, branches, and seeds (tactile); and programs where participants eat food and drink tea (palate) [32].

The activity, season, and location classifications were based on the contents written by the forest healing instructors. For instance, activities were divided into dynamic, static, and dynamic–static activities; seasons were divided into the seasons (four seasons, spring, summer, fall, and winter) or simply all of them if year-round; and lastly, locations were divided into indoor, outdoor, and both indoor–outdoor.

In addition, the classification of forest healing programs included activity types (one day, day and night, or regular), education personnel, and participation fees, whereas the classification criteria were selected based on prior research. When classifying types, if there were one or more characteristics of the activity contents, all of them were included in the type, whereas a frequency analysis of the 268 detailed program activities was conducted according to the six major classifications.

After the frequency analysis, a systematic review (SR) was performed to determine the strength of evidence for forest healing program effects. The detailed programs were then classified according to activity, type, and duration categories.

### 2.2. Effectiveness of Forest Healing Program through SR and Meta-Analyses

#### 2.2.1. ‘Health Benefits of Forests to People’ through SR

An SR was conducted to analyze the activity-specific effects in forests. When selecting each case, the keywords provided in Table 3 were used according to “PICOS.” The keywords were searched in four electronic databases: PubMed, PsycINFO, Web of Science, and Scopus. The present study included papers published in English between January 2000 and February 2021.

**Figure 3:**
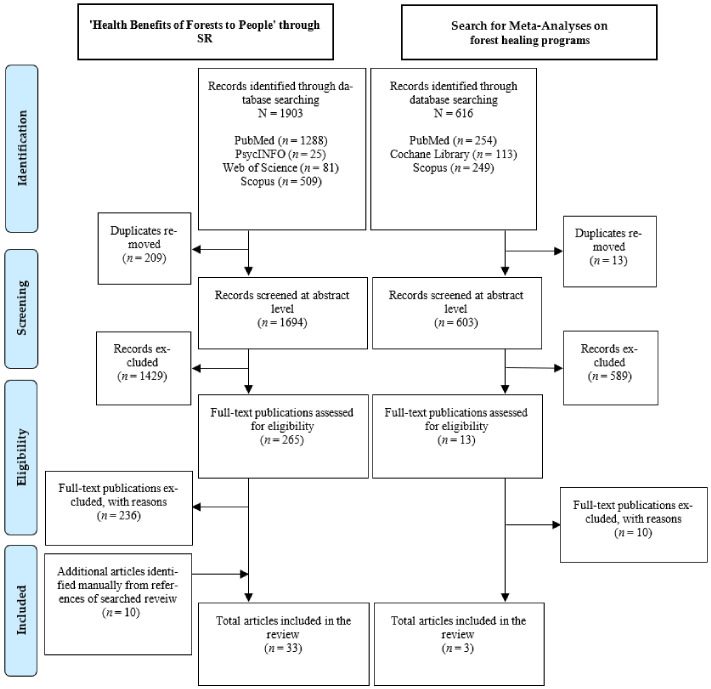
Flow diagram illustrating the selection process.

A total of 1903 search results were exported to the EndNote Citation Manager software (version EndNote X9.3.3. Clarivate Analytics, New York, NY, USA) with 1288 electronic database searches in PubMed, 25 in PsycINFO, 81 in Web of Science, and 509 in Scopus searches. After removing 209 duplicates, the titles and abstracts of the 1694 publications were reviewed. Two reviewers independently screened (EK and GK) the full text for 265 articles, after removing 1429 non-randomized studies or explicitly unrelated subjects, to identify the studies to be included in the systematic literature review. This discrepancy was resolved through discussion. In addition, the final 33 studies were included by reviewing references from existing systematic literature reviews retrieved, adding four qualified studies that were not identified through searches (Figure 3).

To understand the effectiveness of activities in the forest, we also looked at how consistent or mixed positive results have been reported throughout the first round of research, including using the 33 selected studies [34]. The 33 studies were divided into homogeneous groups according to the activities conducted in the intervention. As an indicator for the overall performance for each group, we used an index we called % p + m, which is the ratio of positive outcomes with significant or mixed reports (non-significant) compared to the total number of reports (×100 for percentage). In this study, the p + m ratio was considered to be the effectiveness of activities, and the current status and effectiveness of activities and time by applying the categories of activities classified through frequency analysis and the number of detailed programs over time.

#### 2.2.2. Search for Meta-Analyses on Forest Healing Programs

To understand the effectiveness of forest healing programs, meta-analyses on existing forest healing programs were selected using the SR process. When selecting the paper, the keywords were searched in three electronic databases: PubMed, Cochrane Library, and Scopus, as shown in Table 3, according to the PICOS used by SR. This includes studies published in English until May 2021.

A total of 616 searches were exported to the EndNote Citation Manager software (version Endnote 20) with 254 from PubMed, 113 from the Cochrane Library, and 249 from Scopus searches. After removing 13 duplicates, the titles and abstracts of 603 publications were reviewed. Two reviewers (SK and EK) independently screened the full text of 13 studies, after eliminating 589 non-meta-research or apparently unrelated subjects, to identify the studies to be included in the systematic literature review. Disagreements were resolved through discussion, and the final three studies were included (Figure 3).

In order to understand the effectiveness of forest healing programs, the analysis was based on the confidence interval (95% CI) using the results of the last three selected studies. If the CI value included zero, it was regarded as “No Effect,” whereas if it did not include zero, it was regarded as an “Effect.” This was intended to determine whether the activities carried out in the forest were effective. Duplicate papers were excluded at this point as significant overlap occurred during the forest healing META analysis. Forest healing program activities were divided into the same criteria used earlier, and a total of 63 activities were effective, whereas 5 activities were ineffective in the META paper.

## 3. Results

### 3.1. Classification of Forest Healing Program

#### 3.1.1. Target Groups for Forest Healing Program Development

An analysis of the frequency of subjects according to the disease states confirmed the following hierarchy: normal > addictive > chronic = environmental diseases; with normal disease states accounting for 90.67% of program activities. Therefore, this can generally be seen as a program activity that considers most (nearly all) possible subjects (Table 4). Furthermore, an analysis of the frequency of the target groups confirmed the following hierarchy: teenagers > adults > families > infants = the elderly = the disabled > pregnant women; with 54.67% of teenagers participating in more than half of the program activities.

#### 3.1.2. Healing Factors and Activity Evaluation

According to the frequency analysis of the six major methods, the order of most to least frequent were plant therapy > psychotherapy > exercise therapy > water therapy > climate therapy > diet therapy; plant therapy accounted for 67.63%, whereas diet only accounted for 0.72%. When classifying detailed programs, there were instances where no specific therapeutic approach was used, whereas others that constituted multiple therapies simultaneously (Table 5).

According to the frequency analysis results for the five sensory activities, the most to least frequent were tactile > olfactory > auditory > visual = palate, with tactile sense activities accounting for 34.22% of the detailed program composition. Furthermore, there were also programs that did not include sensory activities in the composition of detailed programs, while others utilized all five senses.

Furthermore, the results of the frequency analysis of activities show that more than half of the activities were dynamic activities, accounting for 59.70%, and the frequency order given as dynamic activities > static activities > dynamic–static activities.

#### 3.1.3. Season and Training Time

According to the frequency analysis of the seasons, the order of highest to lowest frequency by season were summer > spring = fall > winter. There were 45 activities (60.00%) available in spring, summer, and fall, whereas 25 activities (33.33%) were available for all four seasons. This indicates that fewer programs are operated in winter than in other seasons due to environmental restrictions on outdoor activities (Table 5).

The frequency of forest healing programs for sessions >60 min was higher than <60 min, with 74.25% of the detailed programs running for more than 60 min. Among the detailed programs, the minimum program duration was 10 min for one-day programs, and the maximum program duration was 240 min for two-day and one-night programs.

#### 3.1.4. Location for Forest Healing Programs, Type of Activities, Number of Educations, Participation Fee

The results of the frequency analysis of the venue showed that the outdoor area accounted for 57.09% of activities, by order of outdoor > indoor > outdoor–indoor, which indicates that the forest healing program actively utilized the places created in the healing forest (Table 5).

According to the frequency analysis of the visit characteristics, the order was one day visit = day- and night visits > regular visit. Within the day- and night visits type, it was higher in the order of one night and two days (34.67%) than two nights and three days (12.00%). This is the largest number of one-day visits, with the total program time of one-day programs consisting of 2–6 h, and regular visit programs consisting of 3–15 sessions.

In addition, we analyzed the number of education personnel and participation fee items using 75 programs to analyze the content of forest healing program activities. Education personnel in the forest healing program consist of 5–300. Furthermore, 17 programs were free of charge with green subsidies, and besides free programs, the minimum cost was 1000 KRW, whereas the maximum cost was 165,000 KRW.

### 3.2. Effects of Forest Healing Program

#### 3.2.1. Effectiveness of forest healing programs on Activities and Time

An SR was conducted to determine the effectiveness of forest healing activities and to support the level of evidence for forest healing program effects. The duration of sessions was divided into more or less than 1 h, and activities were divided into staying, walking, exercise, and indirect. Staying included viewing, watching, and meditation in one place, and walking included activities such as walking around and observing the surroundings. Exercise included trekking and forest sports. Finally, indirect included natural materials, audiovisual materials, programs that utilize natural materials, and programs that include audio-visual materials (Table 6).

To determine the current status and effectiveness of the forest healing programs currently implemented, it was divided into static and dynamic activities and further detailed activities, and the criteria were the same as those used by SR. According to the analysis, it was higher in the order of walking > indirect > exercise > staying, with walking accounting for 46.27% (Table 7).

**Figure 4:**
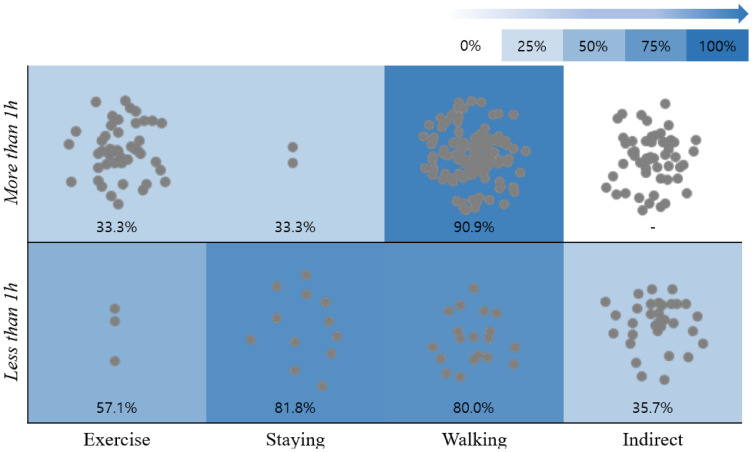
Effect of activities and duration of forest healing program on mental (psychological) health. Background color: The forest healing effect size (% p + m) is expressed as 0~100%, indicating that darker colors have a greater effect on forest healing; gray dots: the detailed forest healing program was classified based on the activities and their duration. Each gray dot in the figure represents the type of activity and its duration for each program.

**Figure 5:**
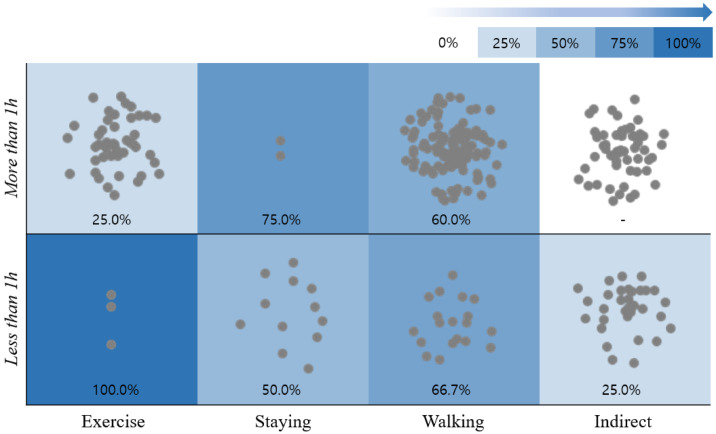
Effect of activities and duration of forest healing program on the physical (physiological) health. Background color: the forest healing effect size (% p + m) is expressed as 0~100%, indicating that darker colors have a greater effect on forest healing; gray dots: The detailed forest healing program was classified by activity and time, and each gray dot represents the type of activity and its duration for each program.

To understand the level of evidence for the effectiveness of forest healing programs, we applied the activity category and time effectiveness of the SR, as well as the activity frequency of programs to illustrate the activity, time, and effectiveness of the ongoing forest healing programs (Figure 4 and Figure 5). The background color represents the activity and time effect (% p + m) after SR, with darker colors having a greater effect on forest healing. The gray dots in the figure represent the activity performed under the detailed forest healing program and its duration—the more gray dots in each session, the greater the number of forest healing programs that conduct the corresponding activity for the mentioned duration.

According to the results, the most psychologically beneficial activity was walking, with 80.0% of sessions <1 h and 90.9% of session >1 h showing positive outcomes (which was also the most frequent activity). Physiological activities are considered to be highly effective exercise for both >1 h and <1 h, each showing great physical effects. However, detailed programs were not actively implemented.

Furthermore, walking activities are both psychologically and physically >60.0% effective, indicating that the level of detailed programming is more active than other activities.

In the case of indirect activities, no studies were available for activities >1 h. Therefore, detailed forest healing programs are currently underway with the effectiveness yet to be evaluated.

These results indicate that for psychological benefits, walking activities work regardless of duration, whereas staying activities is 48.5% more effective for shorter durations than >1 h. This can be explained by a sense of boredom when a static program is run for >1 h. Additionally, % p + m showed that the physiological benefit of >1 h of walking activity is >60% effective, whereas <1 h of exercise is 100% effective. However, the main measurement tool of staying is heart rate variability, which is determined by movement, and this indicator may vary depending on the design of the study because there is a significant difference between measurements with or without sufficient rest.

#### 3.2.2. Evidence for Effectiveness of Forest Healing Programs

We compared the activities of detailed forest healing programs used after certification and the composition of forest healing programs supported by META (Table 8). Walking (132 cases, 44.44%) accounted for most of the activities of detailed forest healing programs being used after certification, with 31 cases also accounting for the most activities confirmed through the META paper. Most activities have been shown to be effective when forest healing occurs, but a total of five studies related to walking showed no effect.

#### 3.2.3. Psychological and Physiological Effects of Forest Healing Programs

**Figure 6:**
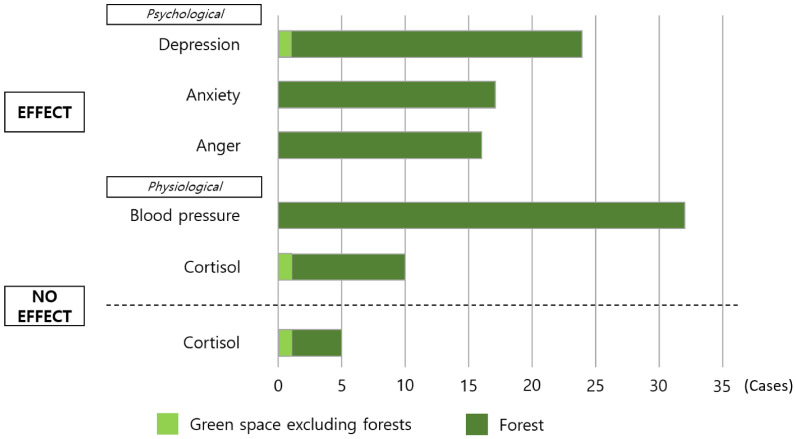
Overview of psychological and physiological outcomes of forest healing programs.

In the final three selected META studies, the results were divided into either effect or no effect through the CI index, and the effectiveness of the forest healing program was identified through research methods conducted in each paper (Figure 6). Most of the experimental sites were forests, but three were conducted in places other than the forest. Among the studies that showed forest healing effects, blood pressure had the highest number of therapeutic effects in the study, with 32 cases, followed by 24 cases for depression, 17 cases for anxiety, 16 cases for anger, and 10 cases related to cortisol. Furthermore, five studies involving cortisol, a stress biomarker, showed no forest healing effects.

## 4. Discussion

Forest healing programs are not only helpful for people who aim to improve their lifestyle but can also be therapeutic in recovering, rehabilitating, treating, and preventing diseases. These government-run facilities play a supplementary role in improving health by utilizing nature as a means of facilitating therapeutic purposes. Currently, primary outcomes (effects) of these programs are verified by assessing the purpose for a specific target group in developing forest healing programs; however, the number of verification studies are limited and biased toward fragmentary effects. In addition, efforts have been made to evaluate the effectiveness of the program by utilizing program satisfaction surveys in the field, but this is insufficient to evaluate health outcomes, or overall facility and operational effectiveness. Therefore, in this study, we performed a meta-analysis of previous research to evaluate the level of quantitative and qualitative evidence for the programs currently in operation in Korea. As there has not been an established basis for evaluating forest healing programs, we analyzed forest healing programs in detail to identify the most common characteristics and variables which determine forest healing effects through systematic review and meta-analysis.

Currently, 90.67% of those who participate in the forest healing programs form part of planned programs for the general public. It can be said that forest healing programs have broader implications considering all subjects rather than only targeting individuals with a specific disease or conducting professional activities only required by a specific target group. This does, however, mean that specialization and advancement of forest healing programs are needed [35]. However, this showed that certified forest healing programs are mainly characterized by youth education, and that these programs are significant in educational activities beyond the aims of “healing”. Among the various therapies used in forest healing, many programs were used to utilize plants, improve mindfulness, and promote health by physical activity, with programs that comprise of tactile activities being the most prominent. In addition, detailed programs were operated using dynamic activities, with walking by far the most common activity. This was the same as the results of the existing study, and the activities conducted in the field or research showed that the verified activities were skewed to one side.

It has been reported previously that a program operated over multiple sessions is more effective than over a single session [36]. However, recurring (continuous) visitations are often difficult and would need to be addressed in future research to improve the benefit of forest healing programs. Forest healing instructors who plan and develop programs have to organize them based on the desired outcomes; however, they still face the difficulties of having insufficient evidence and data to design the best-informed program, and even if the subjects receive the best program for their situation, they are usually reluctant (have to be urged) to participate in regular programs.

The documents have shown that most walking and staying activities, such as meditation for >1 h, are most effective psychologically and physiologically; however, there are far more indoor activities that are less effective because of operational convenience (easier). A review of the quantitative distribution of the ground by disease and activity showed that some studies did not actually show the expected physical changes or did not utilize appropriate indicators, timing, and duration to measure the changes. In addition, if forest healing activities currently conducted for the public become certified for certain diseases (such as depression and blood pressure), it will improve the professionalism of these forest healing programs in the future.

This study aimed to examine the forest healing programs certified in Korea through SR and meta-analysis. We determined the current status of certified forest healing programs and divided them into duration and activities to determine the effectiveness of forest healing. Through rigorous analysis, we identified the criteria for effectiveness and potential complications of domestic forest healing programs. To date, the criteria for forest healing programs are diverse and have not been standardized, thereby allowing independent forest healing programs to be established using other certification systems. However, by classifying forest healing programs based on the results from previous studies, our results are able to accomplish the following: establishing a forest healing program certification system according to criteria such as (1) activity, (2) disease state, (3) duration, (4) location, and (5) environment; classify forest healing programs according to six major methods and which of the five senses are stimulated by activities; and provide insight to improve the quality of existing programs through our critical analysis.

Although various studies are currently being conducted, there are no clear criteria for evaluating the basis due to limitations such as targets, indicators, and periods, which will require continuous efforts to systematically measure health conditions for people using forest healing programs.

## 5. Conclusions

This study analyzed the current status of forest healing programs operating in Korea and evaluated the evidence for their effects. To upgrade, develop, and operate these programs, it is necessary to establish a well-organized system based on the existing quantitative and qualitative effects. Our study reported that the largest number of healing programs were designed for participants without diseases, and included plant therapy, activities with tactile stimulation, and occurred outdoors, for a duration of one day, with sessions longer than 60 min. According to the analysis of forest healing cases, the program operations that were expected to have insufficient or low effectiveness for forest healing were prioritized depending on the on-site conditions. In addition, the medical basis for the statistically significant therapeutic effects found for depression, anger, and hypertension are being investigated. It is imperative that factors be identified and reviewed for patients with high probability for therapeutic response from forest healing program enrollment.

## Figures and Tables

**Table 1:** Certification criteria of each institution for forest healing programs.

Category	Korea Forest Service	Ministry of Gender Equality and Family	Ministry of Environment
Certification System	Forest Education Program	Adolescent Training Activity	Selection of Outstanding Environmental Education Program
Purpose	Providing opportunities to understand the necessity and role of forests and the value of forest environment system.	Providing superior programs to help adolescents to participate by choosing beneficial and safe activities	Providing the environmental education programs that are eco-friendly, high-quality, and safe
Criteria	Total 5 index, 9 items Education program: Composition, Management, Evaluation, Faculty members, Education environment (place and equipment, safety control, hygienics), Activity records, Accommodations	Common Criteria, 6 items Individual Criteria: 3 items with accommodation, 5 without accommodation. Special Criteria: 2 items for high risk, 1 for accommodation as groups, and 3 for non-face-to-face (real-time, contents, task performance)	Total 3 index, 5 items Program (quality, management, evaluation), instructors (certification and arrangement), education activity environment (safety control)
Process	Documentation review Field survey and evaluation report of consulting examination of item and review Consideration and decision Issue of certification	Verification consulting Applying for verification Verification examination Consideration to verify Issue of verification	Pre-consulting (optional) Appointed application Appointed examination Appointed consideration Selection as outstanding program

**Table 2:** Classification of 268 detailed forest healing programs.

Classification Criteria	Classification	Detailed Items
Participants	Disease State	Normal, Chronic, Addictive, Environmental
	Target Group	Infant, Adolescent, Aged, Pregnant, Health-Impaired
The Six Methods		Plant Therapy, Water Therapy, Diet, Psychotherapy, Climate Therapy, Exercise
Season		four seasons, Spring, Summer, Fall, Winter
Sensory Activities		Visual, Olfactory, Auditory, Tactile, Palate
Activities		Dynamic activities, Static activities, Both
Locations		Indoor, Indoor/Outdoor, Outdoor

**Table 3:** Keywords used to search for relevant studies.

Keywords	‘Health Benefits of Forests to People’ through SR	Search for Meta-Analyses on Forest Healing Programs
P	(people OR volunteers OR participants OR subjects OR individuals)	(people OR infant OR adolescent OR adult OR elderly OR pregnant women OR disabled)
I	(“natural environment” OR “green space” OR “nature space” OR “green nature” OR “forest”)	(“green space” OR “forest”)
	AND	AND
	(intervention OR program OR programme OR exposure OR therapy OR recreation OR “physical activity” OR exercise OR activities OR walking OR meditation OR staying)	(healing OR therapy OR program)
C	-	
O	“health” OR “well being” OR “well-being” OR “health promotion” OR “physiological” OR “psychological” OR “mental health” OR “physical health” OR therapeutic	
S	“randomized controlled” OR “RCT”	“meta”

RCT: Randomized Controlled Trials.

**Table 4:** Classification of the participants for the forest healing programs.

Section	Subsection	Participation Frequency	Rate (%)
Disease State	Normal	68	90.67
	Chronic disease	2	2.67
	Addictive disease	3	4.00
	Environmental disease	2	2.67
Target Group	Infant	5	6.67%
	Adolescent	41	54.67%
	Adult	15	20.00%
	Elderly	3	4.00%
	Pregnant women	1	1.33%
	Disabled	3	4.00%

**Table 5:** Characteristics of the session types of forest healing programs.

Categories	Detailed Contents		Frequency (Cnt.)		Ratio (%)
Therapeutic approach	Plant therapy		188		67.63
	Water therapy		16		5.76
	Dietary therapy		2		0.72
	Psychotherapy		35		12.59
	Climate therapy		6		2.16
	Exercise		31		11.15
Total			278		100.00
Sense activities	Visual		36		11.96
	Olfactory		74		24.58
	Auditory		52		17.28
	Tactile		103		34.22
	Palate		36		11.96
Total			301		100.00
Type of physical activity	Static activities		75		27.99
	Dynamic activities		160		59.70
	Dynamic–Static activities		33		12.31
Total			268		100.00
Season	Spring		72		96.00
	Summer		75		100.00
	Fall		72		96.00
	Winter		27		36.00
Session duration	>60 min		69		25.75
	<60 min		199		74.25
Total			268		100.00
Venue	outdoor		153		57.09
	indoor		81		30.22
	Outdoor–indoor		34		12.69
Total			268		100.00
Type of visit	One day visit		35		46.67
	Days and nights visit	2 days/1 night		26	34.67
		3 days/2 nights		9	12.00
	Regular visit		5		6.67
Total			75		100.00

**Table 6:** Health outcomes of different activities associated with forest healing program.

Activities	Mental Health						Physical Health					
	+	+/	/	-	% p	% p + m	+	+/	/	-	% p	% p + m
Staying												
<1 h	6	3	2	-	54.5%	81.8%	-	1	1	-	0.0%	50.0%
>1 h	3	1	8	-	25.0%	33.3%	3	-	1	-	75.0%	75.0%
Walking												
<1 h	23	5	5	2	65.7%	80.0%	5	3	4		41.7%	66.7%
>1 h	5	5	1	-	45.5%	90.9%	27	-	18	-	60.0%	60.0%
Exercise												
<1 h	2	2	3	-	28.6%	57.1%	-	1	-	-	0.0%	100.0%
>1 h	-	2	4	-	0.0%	33.3%	-	1	2	1	0.0%	25.0%
Indirect												
<1 h	3	2	8	1	21.4%	35.7%	1	-	3	-	25.0%	25.0%
>1 h	-	-	-	-	-	-	-	-	-	-	-	-

+ reports with significant positive outcomes; +/reports which included both significant and non-significant positive outcomes;/reports with non-significant effects; - reports with negative outcomes; % p percentage of reports with significant positive outcomes compared to total count; % p + m percentage of reports with positive outcomes (including both significant and non-significant) compared to total count.

**Table 7:** Detailed activity types associated with forest healing programs.

Categories	Detailed Contents	Frequency (Cnt.)	Ratio (%)
Activities	Staying	24	8.96
	Walking	124	46.27
	Exercise	41	15.30
	Indirect (natural materials, audiovisual materials)	79	29.48
Total		268	100.00

**Table 8:** Numbers of references providing evidence of forest healing activities.

Activities		Numbers of References	
		Effect	No Effect
Staying	31 (10.44%)	27	-
Walking	132 (44.44%)	31	5
Exercise	48 (16.16%)	1	-
Indirect	86 (28.96%)	4	-
Total	297 (100%)	63	5

## Data Availability

The datasets generated during and analysed during the current study are available from the corresponding author on reasonable request.
